# Acknowledgement to Professor César Amaury Ribeiro da Costa

**DOI:** 10.1590/2175-8239-JBN-2024-IM001en

**Published:** 2023-11-20

**Authors:** Roberto C. Manfro, Cristina Karohl

**Affiliations:** 1 Universidade Federal do Rio Grande do Sul, Faculdade de Medicina, Porto Alegre, RS, Brazil.; 2 Hospital de Clínicas de Porto Alegre, Porto Alegre, RS, Brazil.

**Figure 1.**
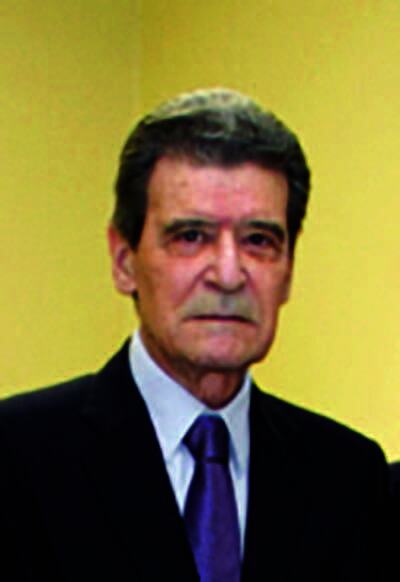
Professor Cesar Costa at the celebration of the 40^th^ anniversary of the HCPA Nephrology Service in 2013.

Writing about Professor César Costa invokes an avalanche of memories and emotions. Perhaps it would be simpler to say that Professor César Costa was, throughout his career, an example to be followed as a doctor, teacher, mentor and citizen (Figure 1). It is impossible not to remember the quality, class and distinction that he lent to every activity in which he was involved, particularly to Nephrology clinical meetings. And that his presence was synonyms with objective, high-level, academic, in-depth systematic, useful discussions. We were his undergraduate students, residents, graduate students, and early on we became admirers and soon enough his friends.

Professor César Costa was born in Lages, Santa Catarina, Brazil, in 1930, where he finished high school in 1948. He attended the School of Medicine of the Federal University of Rio Grande do Sul (UFRGS) between 1950 and 1955. In 1957, the gifted academic was admitted to the teaching staff of the school he attended as a teaching assistant. In subsequent years, he climbed the academic rankings to achieve the position of Full Professor in the Department of Internal Medicine in 1988.

In the mid-1960s, at a time when this was unusual, he sought to improve and challenge himself and attended graduate internships at the Cornell University Medical College, in New York, and at the University of California, in Los Angeles, as a scholarship holder of the American College of Physicians and the Kellogg Foundation. As he returned to Porto Alegre, he became the main catalyst for the exponential development of the young and vibrant specialty that was Nephrology. A visionary, he was among the first Brazilian doctors to publish in the area of kidney transplantation. He wrote several articles and published several chapters in renowned Nephrology textbooks. Some of his main collaborations are listed in the references below^1,2,3,4,5,6,7,8^.

His life in the Academia was immensely rewarding. He was honored many times by students at the School of Medicine at UFRGS and was the patron of seven graduating classes. He created and structured the Nephrology Service at the Hospital de Clínicas de Porto Alegre (HCPA) and was a preceptor for the medical residency program in Nephrology at HCPA opened in 1973. At HCPA, he served as head of the Nephrology Service from 1975 to 1984 and medical director from 1984 to 1988. In 1971, he created the first Graduate Program in Nephrology in Brazil, at UFRGS, where he worked as Coordinator for many years.

In his life as a member of medical associations, Dr. Costa was a founding member of the Brazilian Society of Nephrology (SBN) in 1961, and a member of the Latin American Society of Nephrology and the International Society of Nephrology. He held the position of president of the Department of Urology and Nephrology of the Rio Grande do Sul Medical Association (1962–1963), Vice-President (1978–1980) and President of the SBN (1980–1982), and editor of the Brazilian Journal of Nephrology in 1982. He was a founding member of the Rio Grande do Sul Academy of Medicine and an honorary member of the National Academy of Medicine.

A dedicated and very successful doctor, he was respected and admired by countless patients in his practice. He was married to psychiatrist Flávia de Camargo Costa, with whom he had two children.

However, writing solely about his medical and academic achievements does not do Professor César Costa justice. He died at 92. Countless expressions of grief were uttered on the occasion of his death about his character, correction, elegance, and sensitivity, beyond his immense professional and academic competence. Expressions of admiration such as “in each conversation, a lesson in rationality, simplicity and humanity”, “courtesy framed his immense knowledge”, “a unique example of a master”, “admired and loved by the many who were lucky enough to live with him” and “it was always a joy to meet Professor César, to admire his intelligence and medical and humanistic culture and his cordial, respectful and encouraging behavior with everyone he interacted with. He was a great doctor, teacher and manager, and above all a beautiful human being.”

Professor César Costa was a giant among humans. Like few, he improved everything and everyone around him, innovating and remodeling his surroundings with naturality. For us, who lived with him for many years, and for so many others who had the privilege of knowing him, there remains his legacy, the example, the great memories of a man we miss dearly.
